# Mishaps during intrahospital transport of patients from emergency department - a mixed bag of patients

**DOI:** 10.1186/2197-425X-3-S1-A69

**Published:** 2015-10-01

**Authors:** A Taggu, S Murthy, B Krishna, MKM Varma

**Affiliations:** Critical Care Medicine, St. Johns Medical College Hospital, Bangalore, India; Emergency Medicine, St. Johns Medical College Hospital, Bangalore, India

## Introduction

Intrahospital transport (IHT) of patients from emergency department (ED) is not without risk. Studies on non-intensive care unit (ICU) patients' IHT related mishaps are few.

## Objectives

To observe the types and incidence of mishaps experienced during IHT of both critically ill and non-ICU patients from ED.

## Methods

A prospective observational study of consecutive patients presenting to ED between 1st November 2014 to 27th February 2015 was conducted after institutional ethical committee clearance. Inclusion criteria included all patients requiring transport within or outside the ED for diagnostic or therapeutic interventions and to inpatient sites like ICU.Patients with incomplete data were excluded.

As per the study protocol the escorting ED residents completed the prescribed data sheet for critically ill patients during or immediately after the transport. For escorting non-ICU patients (clinically less unstable), a ED nurse or sometimes untrained personnels were deputed. Few patients had multiple transports but since these IHT differed in destination, duration and nature, each was recorded as an independent event. The mishaps were grouped into equipment related, patient related, line related and miscellaneous groups[1].

## Results

Total of two hundred and six patients (245 transport events) were enrolled with 46.6% (96/206) critically ill patients and 53.3% (110/206) non-ICU patients. 72.6% (178/245) of transports were associated with one hundred and twenty IHT mishaps. Maximum mishaps were equipment related ( 70/120; 58.3%, 95% CI = 52.3%- 62.4%). Of these, Oxygen saturation probe displacement, ECG lead displacement, power cord tangle and infusion interruptions were the main causes. Patient related mishaps (30/120, 25%, 95% CI= 21.6%- 28.4%) mainly consisted of hypotension and desaturation needing interventions like fluid resuscitation and oxygen supplementation. Line related mishaps (14/120, 11.6%, 95% CI= 9.8%- 14.5%) were intravenous line (IV) tangle and arterial line kinking. Transport delay and non-availability of beds were the main miscellaneous causes (6/120,5%,95%CI= 3.8%-7.6%). In the non-ICU group, on the way to medical ward, two patients had significant hypotension (gastroenterirtis) needing IV fluid resuscitation and prompt ICU admission after ED resident intervened. One had altered sensorium (hypoglycemia) and was corrected with oral glucose. Though overall mishaps were lesser among non-ICU patients, late recognition of symptoms could have been fatal.

## Conclusions

Mishaps during IHT of patients from ED is common and potentially dangerous. Adherence to IHT protocols and need for formal guidelines for transport of non-ICU patients is recommended.
